# Mechanism of Lysoforte in Improving Jejuna Morphology and Health in Broiler Chickens

**DOI:** 10.3389/fvets.2022.946148

**Published:** 2022-07-19

**Authors:** Xiaofeng Li, Xiaoli Shi, Noura M. Mesalam, Lei Liu, Zhihao Chen, Bing Yang

**Affiliations:** ^1^College of Animal Science, Anhui Science and Technology University, Fengyang, China; ^2^College of Animal Science, Guizhou University, Guiyang, China; ^3^Biological Applications Department, Nuclear Research Center, Egyptian Atomic Energy Authority, Abu-Zaabal, Egypt; ^4^Center of Reproductive Medicine, The First Affiliated Hospital of Bengbu Medical College, Bengbu, China

**Keywords:** gene, signaling pathway, Lysoforte, jejuna, broiler

## Abstract

Lysoforte (LFT) plays a vital role in maintaining broilers' health and intestinal morphology. However, the mechanism behind the effects of LFT improving intestinal morphology and health is still unclear. Therefore, this study was implemented to explore the central genes linked to the regulatory effect of LFT. Seventy-five newly hatched Cobb 500 male broilers were randomly divided into three groups: control, LFT500, and LFT1000 groups, with 25 chicks per group. The control chicks were provided with the basal diet, and the birds in LFT500 and LFT1000 groups were offered the same basal diet with 500 g/ton and 1,000 g/ton LFT, respectively. GSE94622 dataset consisted of the control and two LFT-treated groups (LFT500 and LFT1000). Jejuna samples were obtained from Gene Expression Omnibus (GEO). Totally 106–344 DEGs were obtained by comparing LFT500 and LFT1000 vs. control and LFT1000 vs. LFT500. Gene ontology (GO) enrichment suggested that the DEGs are mainly related to the phosphatidylethanolamine biosynthetic process and neuron projection extension. KEGG analysis suggested the DEGs were enriched in AGE-RAGE, fatty acid elongation, ECM-receptor interaction (ECMRI), glycerophospholipid metabolism, focal adhesion, unsaturated fatty acids biosynthesis, and ABC transporters. Moreover, 29 genes, such as *REG4, GJB1, KAT2A, APOA5, SERPINE2, ELOVL1, ABCC2, ANKRD9, CYP4V2*, and *PISD*, might be closely related to promoting jejuna morphology in broilers. Taken together, our observation enhances the understanding of LFT in maintaining intestinal architecture and the general health of broiler chickens.

## Introduction

Fats and oils, which are the most important dietary sources of energy, are excellent ways to accumulate the energy requirements for the optimized weight gain of broiler chickens (1, 2). Exogenous emulsifiers played an effective role in improving lipids utilization in broiler chickens, as the latter fail to gain lipids due to their poor emulsification in the gut (3). Lysoforte (LFT), a lysolecithin produced from lecithin that acts as an efficient emulsifier, could improve poultry growth, reproduction, and carcass quality by enhancing nutrient digestion and absorption, as well as reducing their mortality. In broiler chickens, LFT supplementation elevated average daily gain (ADG), final body weight, relative growth rate, dressing percent, carcass quality, net profit, total return, economic efficiency, and reduced energy matrix value, FCR, and mortality rate (4, 5). Previous research has shown that LFT supplementation increased saturated fatty acids absorption. In addition, a synergistic effect has existed between LFT and enzymes in broilers (6). Papadopoulos et al. (7) reported that fat digestibility, digesta viscosity, and apparent metabolizable energy in chicken were improved by LFT supplementation.

LFT may also have a vital role in maintaining intestinal morphology and health in broilers. LFT supplementation decreased the mucosal thickness at 28 day and induced alterations in the duodenum morphology (7). LFT addition significantly increased the average villus length and width (8). LFT addition had the potential to improve the chicken jejunal morphology and health due to the changes inducted by LFT in the intestinal epithelium (8). However, the mechanism by which LFT improves intestinal morphology and health in broilers is unclear. Therefore, we obtained the microarray data of broiler jejuna treated with or without LFT from the GEO dataset (https://www.ncbi.nlm.nih.gov/geo/) and identified differentially expressed genes (DEGs) in birds' jejuna, aiming to explore the mechanism behind the regulation of LFT on the jejuna morphology and health in broilers.

## Materials and Methods

### Ethics Statement

The present study was approved by the protocol from Anhui Science and Technology University (Bengbu, China) Institutional Animal Care and Use Committee (ECASTU-2015-P08).

### Animals, Feed, and Tissue Collection

Seventy-five newly hatched Cobb 500 male broilers were randomly divided into three groups: control, LFT500, and LFT1000 groups, with 25 chicks per group. The control chicks were provided with the basal diet, and the birds in LFT500 and LFT1000 groups were offered the same basal diet with 500 and 1,000 g/ton LFT, respectively (8). The study lasted for 4 weeks. Ingredients and nutrient contents of the basal diet are presented in Supplementary Table 1. All birds were placed in the room with adjoining floor pens and weighed individually per week (8). On test day 10, five chicks per group were randomly chosen and killed *via* cervical dislocation. Pieces of ~10 cm in length were collected from the middle of the jejuna (8).

### RNA Extraction and Microarray Analysis

Given the LFT effects on chicken jejunal morphology (Supplementary Table 2), ~50 mg of jejuna mucosa was homogenized with Tri Reagent (8). Total RNA was extracted using Directzol RNA columns, and the quality, purity, and integrity of RNA were assessed (8). Microarray analysis was performed with the chicken genome 1.0 array (8). The jejuna gene expression data were deposited in GEO (accession number: GSE94622) (8).

### Microarray Data

GSE94622 consisted of the control (*n* = 5; GSM2479490, GSM2479491, GSM2479506, GSM2479507, and GSM2479523), LFT500-treated (*n* = 5; GSM2479533, GSM2479517, GSM2479516, GSM2479501, and GSM2479500), and LFT1000-treated (*n* = 5; GSM2479503, GSM2479520, GSM2479521, GSM2479537, and GSM2479536) broiler jejuna samples obtained at the 10th day of the experiment.

### Data Processing

To obtain the DEGs between the jejuna samples treated with and without LFT, GEO2R (http://www.ncbi.nlm.nih.gov/geo/geo2r) was used to analyze the data from GSE94622. DEGs were identified as the genes with |log2-fold change (FC)| > 1 and *P* < 0.05.

### Analysis of KEGG and Genetic Ontology for DEGs

KOBAS 3.0 (http://kobas.cbi.pku.edu.cn/kobas3/genelist/) was used to analyze the signaling pathways for DEGs. Regarding the genetic ontology (GO) analysis, DEGs were analyzed with DAVID (https://david.ncifcrf.gov/).

### Protein Classification and Reactome Analysis for DEGs

Protein classification and Reactome analysis for DEGs were performed with the PANTHER (http://pantherdb.org/) and KOBAS 3.0.

### Protein–Protein Interaction

STRING (https://string-db.org/) was employed to form protein–protein interaction (PPI). Cytoscape (version 3.8.0, http://www.cytoscape.org/) was used for further visualization.

### Hub Genes and Their Functions

CytoHubba (http://apps.cytoscape.org/apps/cytohubba) was used to reveal hub genes from the PPI network, then the hub gene functions were summarized using GeneCards (https://www.genecards.org/), previous reports, and NCBI (https://www.ncbi.nlm.nih.gov/).

## Results

### The Outline of Transcripts and Genes in Broilers Jejuna

**Figure 1:**
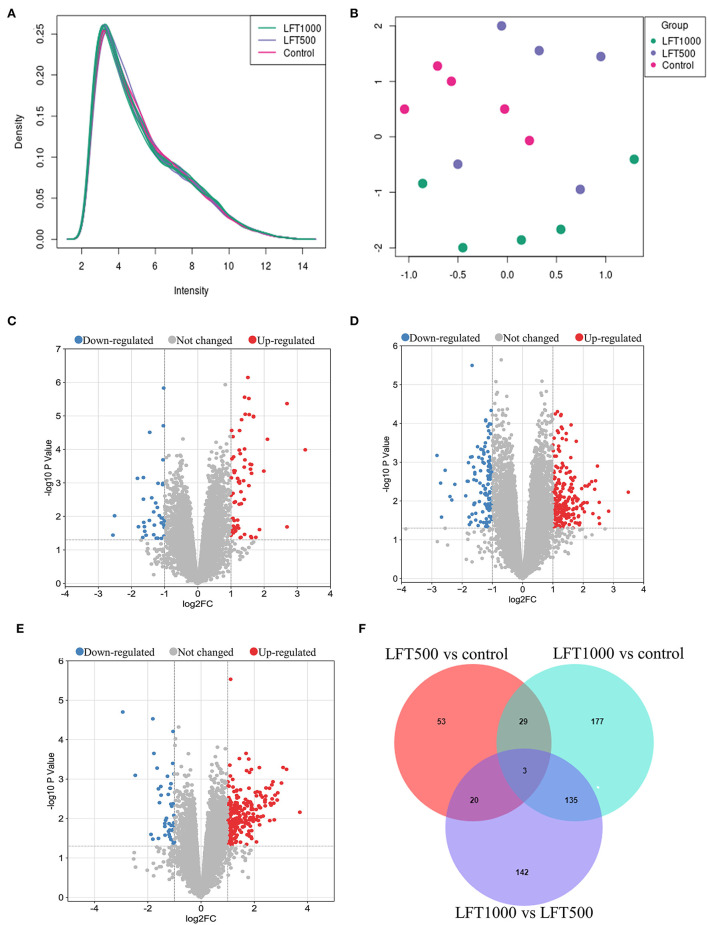
Transcript and Gene Profiles in Broilers Jejuna Treated with and without LFT. **(A)** The density of transcripts expression; **(B)** UMAP; **(C–E)** Volcano plot of DEGs was identified by three comparisons (LFT500 and LFT1000 vs. control and LFT1000 vs. LFT500, respectively). The red, gray, and blue spots represent the upregulated, unchanged, and downregulated genes. **(F)** Venn diagrams for the DEGs are identified in the three ways of comparisons mentioned above.

A total of 38,535 transcripts and 14,086 genes were observed in the chicken jejuna. Transcripts expression density and UMAP are indicated in Figures 1A,B. Figures 1C–E represents the volcano plots for DEGs in the three comparisons of LFT500 and LFT1000 vs. control and LFT1000 vs. LFT500, respectively. The jejuna diagram for DEGs in the three comparisons mentioned above is shown in Figure 1F. As shown in Supplementary Table 3, a total of 174–547 differentially expressed transcripts (DETs) and 106–344 DEGs were identified by the three comparisons. Compared with the control jejuna, 311 transcripts and 224 genes were upregulated, while 236 transcripts and 120 genes were downregulated in LFT1000-treated jejuna (Additional Files 1, 2); 98 transcripts and 68 genes were upregulated, while 76 transcripts and 38 genes were downregulated in LFT500-treated jejuna (Additional Files 3, 4). The top 20 genes up- and downregulated in the three comparisons (LFT500 and LFT1000 vs. control and LFT1000 vs. LFT500) were revealed in Supplementary Tables 4–9.

Twenty-nine common DEGs from the two comparisons (LFT1000 and LFT500 vs. control) are shown in Supplementary Table 10. These genes (including *REG4, GJB1, KAT2A, APOA5, SERPINE2, ELOVL1, ABCC2, ANKRD9, CYP4V2, PISD, PTGR1*, and *AKAP9*) might be closely related to promoting the jejuna morphology and health in broilers.

### GO Analysis for DEGs

**Figure 2:**
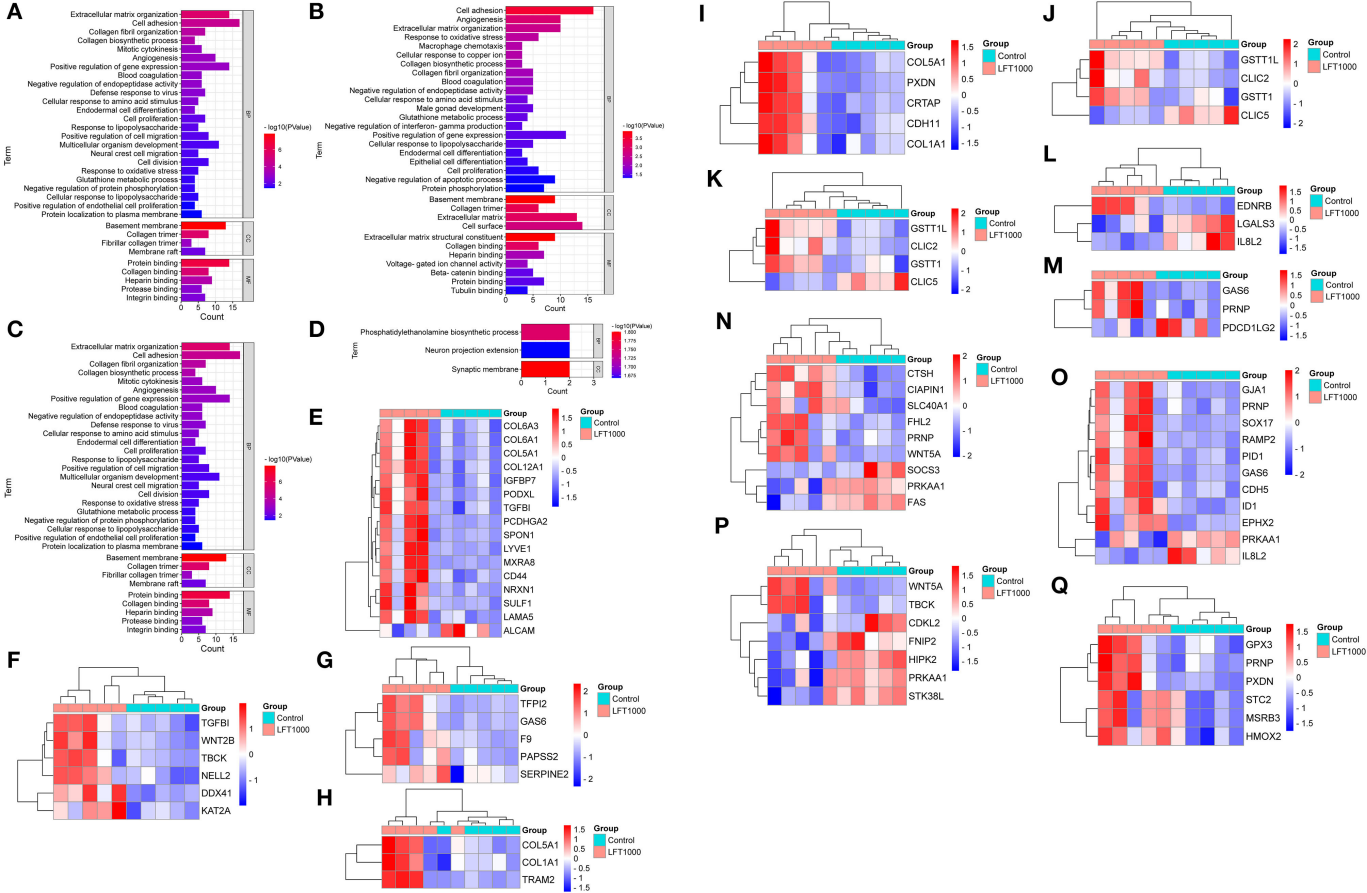
GO Enrichment for DEGs in Broilers Jejuna Treated with and without LFT. **(A–C)** GO enrichment for DEGs identified in the three comparisons (LFT500 and LFT1000 vs. control and LFT1000 vs. LFT500, respectively). **(D)** GO enrichment for the common DEGs in the two comparisons (LFT500 and LFT1000 vs. control). **(E–Q)** The heatmaps for DEGs in cell adhesion, cell proliferation, blood coagulation, collagen biosynthetic process, collagen fibril organization, glutathione metabolic process, macrophage chemotaxis, negative regulation of interferon-gamma production, positive regulation of gene expression, protein phosphorylation, and the response to oxidative stress.

To reveal the biological processes associated with LFT regulation on broiler jejuna, GO analysis of DEGs in the three comparisons, including LFT1000 and LFT500 vs. control, and LFT1000 vs. LFT500 was illustrated in Figures 2A–C and Additional Files 5–7, respectively. DEGs obtained by comparing LFT1000 vs. control may participate in multiple biological processes, such as angiogenesis, blood coagulation, macrophage chemotaxis, glutathione metabolic process, collagen biosynthetic process, extracellular matrix (ECM) organization, the response to oxidative stress, and cell adhesion, proliferation, and differentiation.

DEGs between LFT1000 and the control may have a vital role in protein phosphorylation; leukocyte activation; adaptive immune response; antibacterial humoral response; inflammatory response; innate immune response; osteoclast differentiation; the negative regulation of viral genome replication and apoptotic process; and the positive regulation of production of chemokine, interferon-beta, interleukin-6, tumor necrosis factor; and the positive regulation of NIK/NF-kB pathway.

Twenty-nine common DEGs of two comparisons (LFT1000 and LFT500 vs. control) in the chicken jejuna were closely associated with phosphatidylethanolamine biosynthetic process and neuron projection extension (Figure 2D). In addition, Figures 2E–Q represents the heatmaps for DEGs in cell adhesion, cell proliferation, blood coagulation, collagen biosynthetic process, collagen fibril organization, glutathione metabolic process, macrophage chemotaxis, the negative regulation of interferon-gamma production, and the positive regulation of gene expression, protein phosphorylation, and the response to oxidative stress, respectively.

### KEGG Enrichment for DEGs

**Figure 3:**
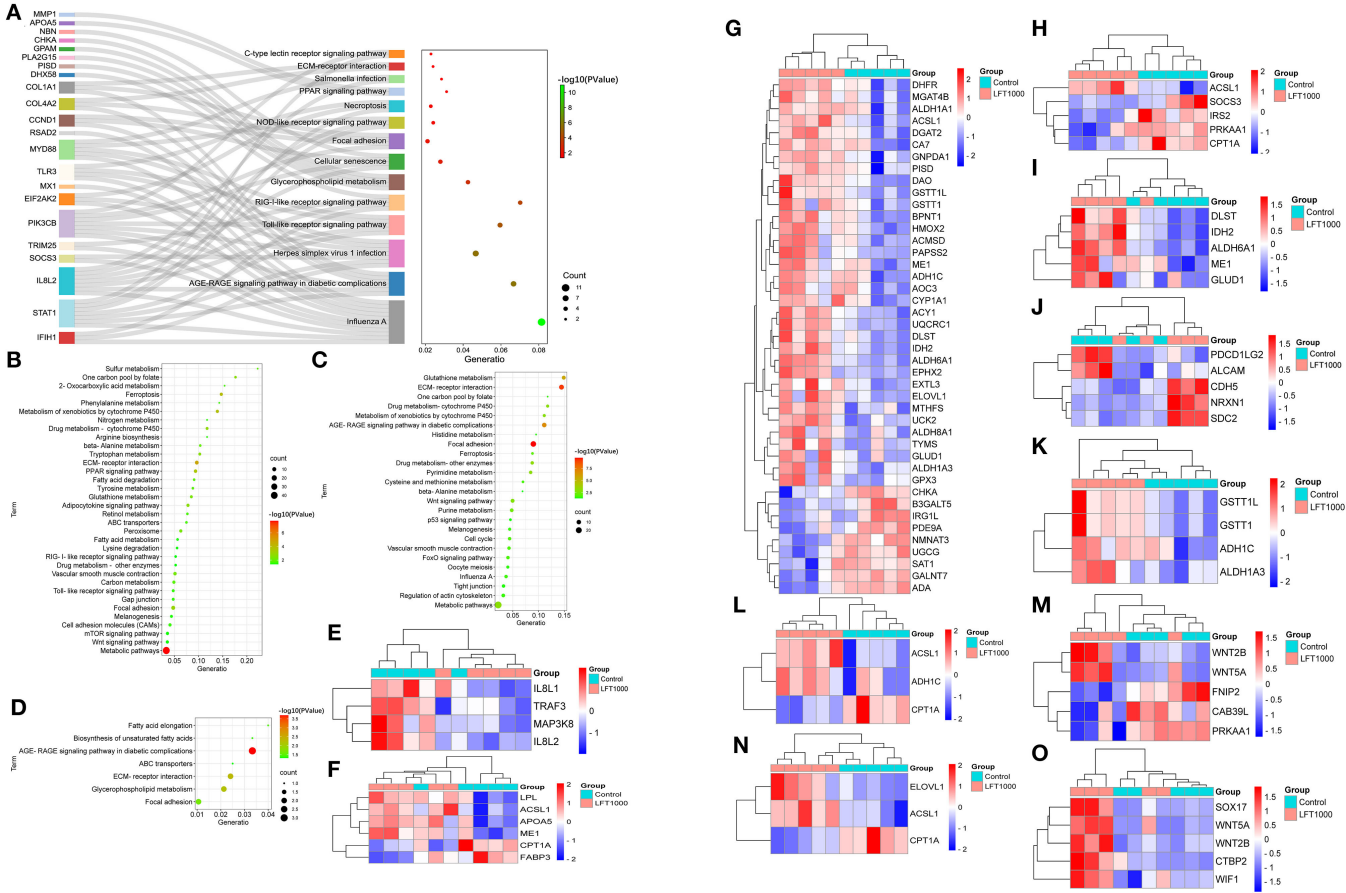
KEGG Enrichment for DEGs in Broilers Jejuna Treated with and without LFT. **(A–C)** KEGG enrichment for DEGs was identified by three comparisons (LFT500 and LFT1000 vs. control and LFT1000 vs. LFT500, respectively). **(D)** KEGG enrichment for the common DEGs was identified by two comparisons (LFT500 and LFT1000 vs. control). **(E–O)** Heatmaps for DEGs in TLR, PPAR, metabolism, adipocytokine, carbon metabolism, CAMs, drug metabolism-cytochrome P450, fatty acid degradation, mTOR, fatty acid metabolism, and Wnt signaling pathway.

To discover the pathways related to the regulation of LFT on broiler intestinal morphology and health, DEGs in the three comparisons, including LFT500 vs. control, LFT1000 vs. control, and LFT1000 vs. LFT500, were implemented in KEGG analysis, and the results were shown in Figures 3A–C, respectively (Additional Files 8–10). As illustrated in Figure 3D, the common DEGs of two comparisons (LFT500 and LFT1000 vs. control) are mainly linked to ABC transporters, AGE-RAGE, fatty acid elongation, focal adhesion, glycerophospholipid metabolism, ECMRI, and unsaturated fatty acids biosynthesis pathways. In addition, Figures 3E–O reveals toll-like receptor (TLR), PPAR, metabolism, adipocytokine, carbon metabolism, cell adhesion molecules, drug metabolism-cytochrome P450, fatty acid degradation, mTOR, fatty acid metabolism, and Wnt signaling pathway, respectively.

### Reactome Enrichment and Protein Classification for DEGs

**Figure 4:**
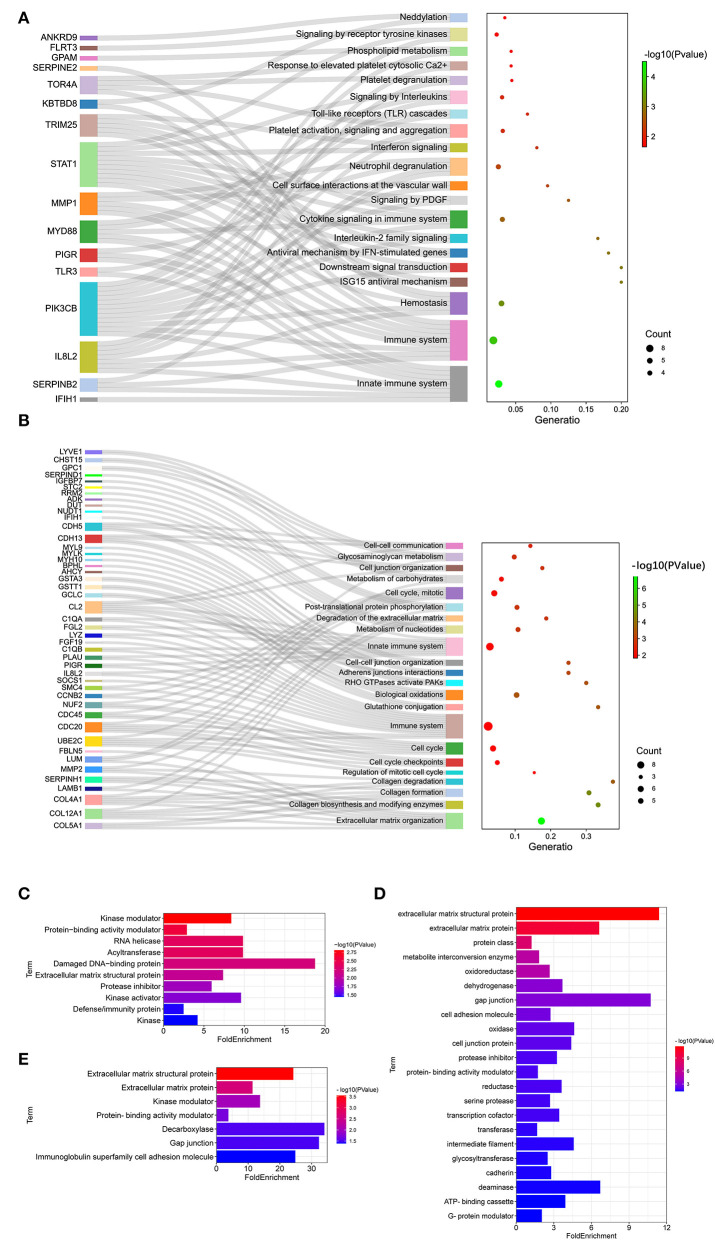
Reactome Analysis and Protein Classification for DEGs. **(A,B)** Reactome analysis for DEGs was identified in two comparisons (LFT1000 and LFT500 vs. control). **(C–E)** Protein classification for DEGs was identified in two comparisons (LFT500 and LFT1000 vs. control and LFT1000 vs. LFT500, respectively).

To further reveal the pathways related to LFT regulation on broiler jejuna heath, Reactome enrichment for DEGs in the three comparisons was performed. DEGs identified by comparing LFT1000 vs. the control may link to the metabolism, hemostasis, signal transduction, small molecules transport, carbohydrates metabolism, platelet activation, glycosaminoglycan metabolism, ECM organization, and neuronal system (Figure 4A). DEGs between LFT500 and the control related to hemostasis, signal transduction, cytokine signaling, neutrophil degranulation, TLR cascades, interleukin-2 family signaling, and platelet activation, signaling, and aggregation (Figure 4B).

Protein classification for DEGs in the three comparisons was performed. DEGs between LFT500 and the control groups might play an important role in acyltransferase, RNA helicase, kinase modulator, protease inhibitor, kinase activator, damaged DNA-binding protein, protein-binding activity modulator, ECM structural protein, and defense/immunity protein kinase (Figure 4C). DEGs between LFT1000 and the control groups might contribute to reductase, oxidase, cadherin, deaminase, transferase, dehydrogenase, oxidoreductase, glycosyltransferase, gap junction, transcription cofactor, serine protease, intermediate filament, protease inhibitor, cell adhesion molecule, ECM structural protein, ECM protein, metabolite interconversion enzyme, cell junction protein, protein-binding activity modulator, and ATP-binding cassette G-protein modulator (Figure 4D). The common DEGs of two comparisons (LFT500 and LFT1000 vs. control) might link to decarboxylase, kinase modulator, gap junction, ECM protein, ECM structural protein, and protein-binding activity modulator, and immunoglobulin superfamily cell adhesion molecule (Figure 4E).

### PPI Network

**Figure 5:**
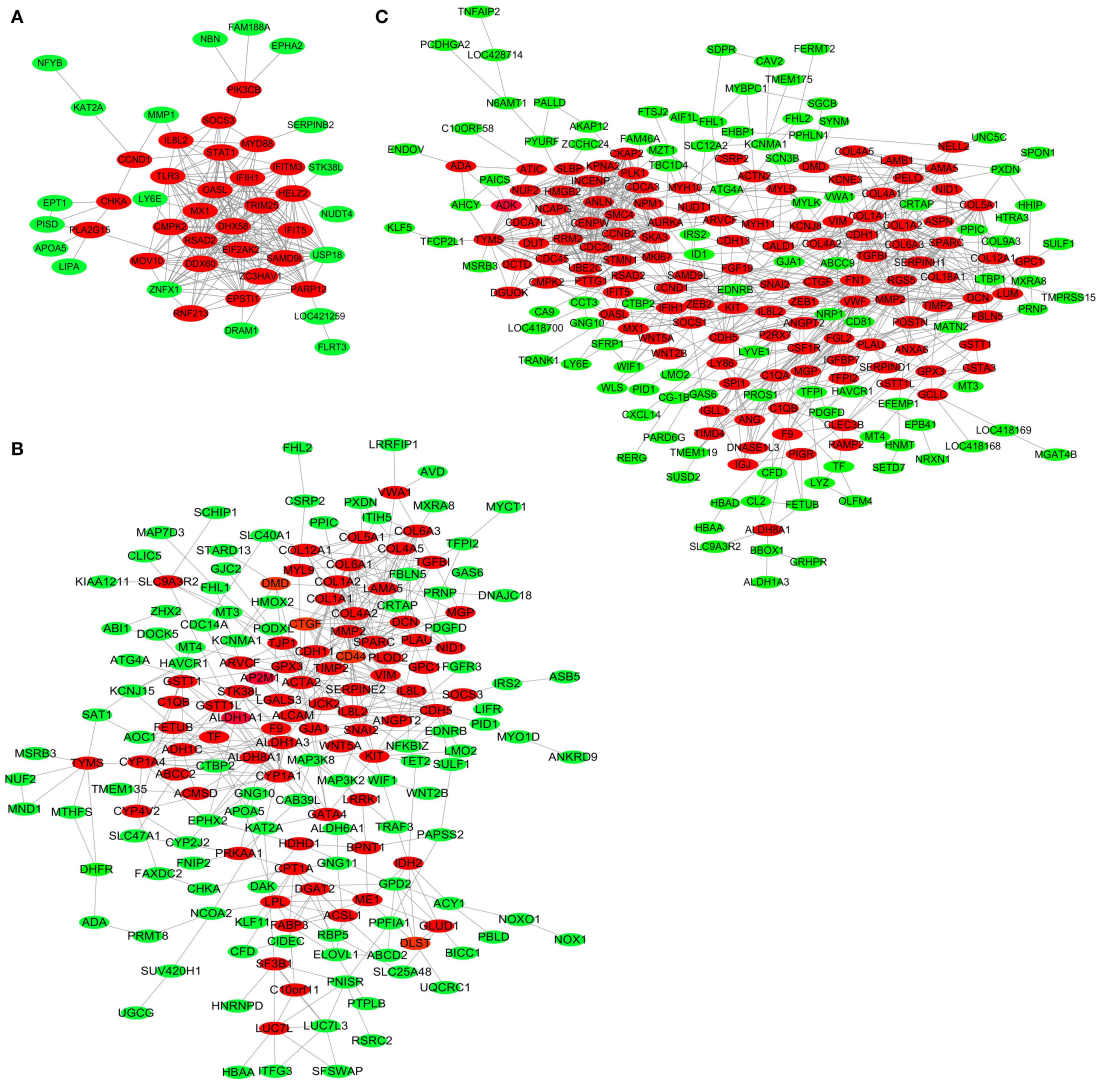
PPI Network for DEGs in broilers jejuna treated with and without LFT. **(A–C)** PPI network for DEGs was identified by three comparisons (LFT1000 and LFT500 vs. control and LFT1000 vs. LFT500, respectively).

To further explore key genes, DEGs in the three comparisons, including LFT500 and LFT1000 vs. control and LFT1000 vs. LFT500, were implemented in PPI networks analysis, and the results are shown in Figures 5A–C, respectively.

### Hub Genes and Their Function

**Figure 6:**
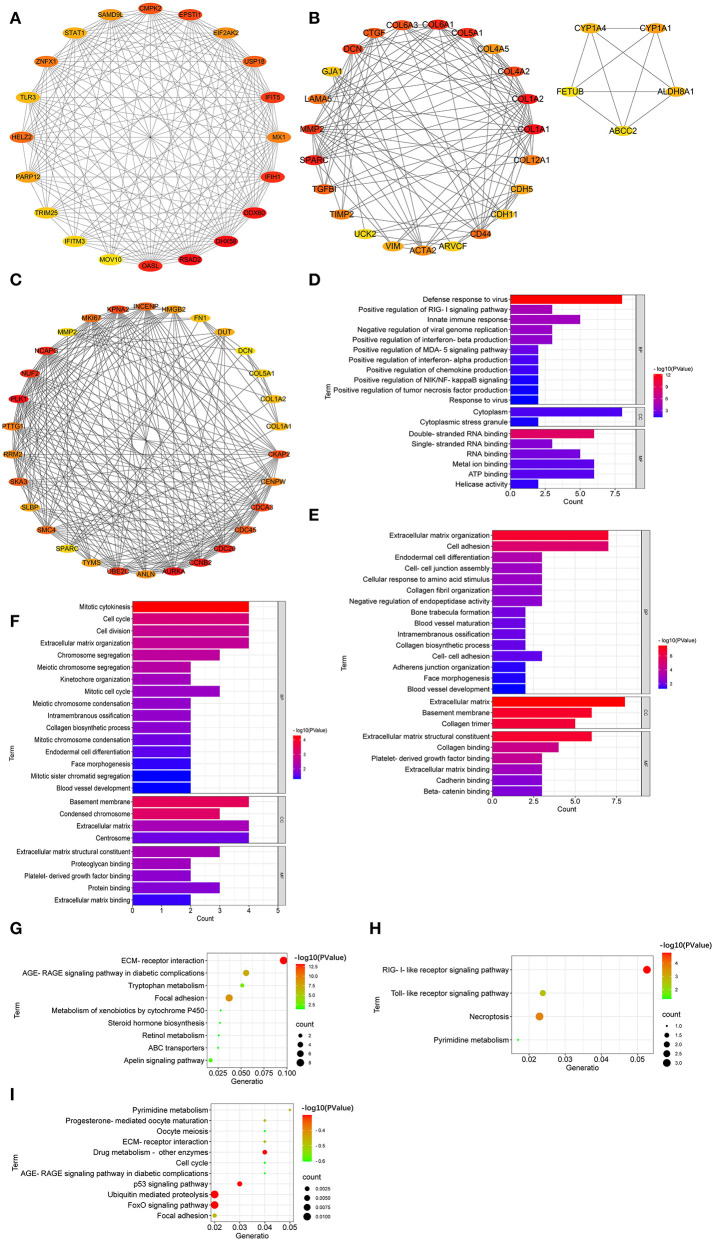
Hub Genes Linked to the Regulation of LFT on the Morphology and Health in Broiler Jejuna. **(A–C)** Hub genes from DEGs were identified by three comparisons (LFT500 and LFT1000 vs. control and LFT1000 vs. LFT500, respectively). **(D–F)** GO and **(G–I)** KEGG enrichment for hub genes mentioned above.

The top 20 hub genes from DEGs between LFT500 and control groups included *RSAD2, DHX58, DDX60, OASL, IFIH1, IFIT5, EPSTI1, CMPK2, USP18*, and *HELZ2* (Figure 6A). The top 28 hub genes from DEGs between LFT1000 and control groups included *COL1A1, COL1A2, SPARC, COL6A1, COL5A1, MMP2, DCN, COL4A2, COL6A3*, and *CTGF* (Figure 6B). The top 30 hub genes from DEGs between LFT1000 and LFT500 groups included *PLK1, AURKA, CCNB2, CDC20, NCAPG, NUF2, UBE2C, CDCA3, KPNA2*, and *CKAP2* (Figure 6C).

GO enrichment suggested that the top 20 hub genes in the comparison of LFT500 vs. control related to innate immune response; the positive regulation of the production of chemokine, interferon-alpha, interferon-beta, and tumor necrosis factor; and the positive regulation of MDA-5, NIK/NF-kB, and RIG-I signaling pathway (Figure 6D). The top 28 hub genes from DEGs between LFT1000 and control groups participated in cell adhesion, endodermal cell differentiation, cell–cell junction assembly, intramembranous ossification, ECM organization, collagen fibril organization, blood vessel maturation, collagen biosynthetic process, blood vessel development, and the negative regulation of endopeptidase activity (Figure 6E). The top 30 hub genes from DEGs between LFT500 and LFT1000 groups are linked to cell division, cell cycle, mitotic cytokinesis, intramembranous ossification, kinetochore organization, chromosome segregation, blood vessel development, mitotic cell cycle, endodermal cell differentiation, mitotic chromosome condensation, collagen biosynthetic process, meiotic chromosome condensation, ECM, mitotic sister chromatid segregation, etc (Figure 6F).

KEGG enrichment suggested that the top 20 hub genes from DEGs between LFT500 and control involved in RIG-I-like receptor, pyrimidine metabolism, necroptosis, and TLR pathways (Figure 6G). The top 28 hub genes compared to LFT1000 vs. control linked to tryptophan metabolism, focal adhesion, retinol metabolism, ABC transporters, AGE-RAGE, apelin, and xenobiotics metabolism by cytochrome P450 signaling pathways (Figure 6H). The top 30 hub genes from DEGs between LFT1000 and LFT500 groups participated in p53, Fox O, AGE-RAGE, cell cycle, focal adhesion, drug metabolism, pyrimidine metabolism, and ECMRI signaling pathways (Figure 6I).

## Discussion

### Hub Genes by Which LFT Maintains the Intestinal Morphology and Health in Broilers

In this study, multiple hub genes, such as *REG4, KAT2A, APOA5, SERPINE2, ELOVL1, ABCC2, ANKRD9, CYP4V2*, and *PISD1*, might participate in the regulation of LFT on intestinal morphology and health in broilers. For instance, *REG4*, a member of the small secretory protein family, was reported to participate in inflammatory bowel diseases and intestinal cancers (9–12).

In our study, 500 and 1,000 g/ton LFT treatment increased the *REG4* expression in the chicken jejuna (2.36- and 2.60-fold), which is consistent with Qi et al. who demonstrated that *REG4* was involved in membrane attack complexes killed inflammatory *Escherichia coli* (*E. coli*) to maintain gut health, and *REG4* gene knockdown increased the content of *E. coli* in the intestinal tract (9). *REG4* was obviously upregulated in colorectal cancer (CRC) tissue compared to the normal tissue. *REG4* expression in CRC tissue was linked to distant and lymph-node metastasis and histologic grade. *REG4* expression in CRC patients showed a worse prognosis (10). REG4 was the biomarker to predict concurrent chemoradiotherapy resistance in patients with rectal cancer. Previous research showed that the significant upregulation of the *REG4* gene was closely related to the undesirable outcome and the aggressive phenotype in rectal cancer patients (11). In our study, *REG4* played an important role in cell regeneration and proliferation. *REG4* expression was linked to higher overall survival and favorable clinicopathological parameters in CRC patients (12).

*KAT2A*, named lysine acetyltransferase 2A, inhibited the proliferation and growth of the intestinal cell, especially in CRC cells. *KAT2A*, succinylate, and succinyltransferase could decrease the α-KGDH complex entered the nucleus, reduce the gene expression, and inhibit the cell proliferation and growth in intestinal cancers (13). Histone acetyltransferase *KAT2A* could interact with long noncoding RNA LBX2-AS1 and RNA-binding protein PTBP1 and regulate the cell proliferation and invasion in CRC (14).

In humans and animals*, APOA5* was a vital gene for intestine chylomicron production and lipids metabolism. *APOA5* decreased the serum triglyceride (TG) by restraining ANGPTL3/8-mediated lipoprotein lipase inhibition (15). Variants in the *APOA5* gene affected TG concentrations and the entire lipoprotein subclass distribution and caused hypertriglyceridemia (16). Hypermethylation in exon 3 of the *APOA5* gene had a positive correlation with the lipoprotein profile and TG concentration linked to atherogenic dyslipidemia. The highest TG concentrations were observed in carriers with a high methylation percentage in the exon 3 of the *APOA5* gene (17). In our study, *APOA5* expression was obviously improved 1.65- and 2.05-fold in chicken jejuna by 500 and 1,000 g/ton LFT treatment, which was consistent with the previous finding that *APOA5* might control TG synthesis and secretion in the intestine. In the TC-7 cell line, saturated fatty acids stimulation obviously increased the *APOA5* gene expression; Similarly, fatty acid butyrate administration improved *APOA5* expression by ~4 times; PPARα agonist treatment also enhanced the *APOA5* expression by 60% (18). In addition, PPARα has a vital role in lipid metabolism and improves ketogenesis and oxidation of fatty acid. PPARα activation reduced food intake and improved insulin sensitivity. Wy-14643 administration significantly increased the expression of *HMG-CoAS2* and *CPT1A* genes in the jejunum. The induction of *HMG-CoAS2* and *CPT1A* expression in the jejunum was linked to the decreased content of lipid droplet. *HMG-CoAS2* and *CPT1A* were two important enzymes for ketogenesis (19).

*SERPINE2* might be a vital gene for intestinal health and disease, such as colorectal cancer. Intestinal epithelial cells (IECs), activation of oncogenic extracellular signal-related kinase (ERK), Ras, or BRAF strongly upregulated the SERPINE2 protein expression and secretion (20). *SERPINE2* gene expression was also dramatically increased in CRC cells compared with normal IECs; In the HCT116 cell, *SERPINE2* knockdown distinctly decreased the anchorage-independent growth, tumor formation, and cell migration in nude mice; *SERPINE2* mRNA level in CRC cell lines was markedly decreased by U0126 (a highly specific MEK1/2inhibitor) administration (21).

*ELOVL1*, a widely expressed gene in tissues from the ELOVL family, encoded the fatty acid elongase to produce C20–C28 fatty acids. In this study, *ELOVL1* was observably upregulated in chicken jejuna from LPC-treated groups, consistent with the report that *ELOVL1* regulated the very-long-chain fatty acid and sphingolipids synthesis, and was closely associated with the intestinal barrier function (22, 23). *ELOVL1* expression induced by inhibiting mTOR1 decreased fatty acids synthesis (22, 23). A previous study in mice indicated that *ELOVL1* knockout induced the defects in the epidermal barrier and the death after birth. In the epidermis of *ELOVL1* knockout mice, the content of C24 sphingomyelin was reduced, but the C20 sphingomyelin level was increased (23).

*ABCC2*, the gene encoding multidrug resistance protein 2 (MRP2), was located on the small intestinal epithelial brush border membrane. *ABCC2* had a vital role in regulating the absorption of nutrients and toxins (24–26). In this study, *ABCC2* expression was dramatically improved in chicken jejuna from LPC-treated groups. This result agreed with the report that *ABCC2* could limit the absorption of toxins, improve the endogenous xenobiotics and substances efflux, and mediate the beneficial effect of *Lactobacillus plantarum* on poultry intestines (24). The expression pattern of MRP2/ABCC2 in the small intestinal tract was tightly regulated. MRP2/ABCC2 expression in the small intestine was closely associated with ezrin phosphorylation status (25). *ABCC2, ABCC3*, and *ABCG2* were expressed in the intestine and could transport the glucuronidated compounds. *ABCC2* knockout significantly decreased the biliary excretion in mice (26). The exposure to thymeleatoxin reduced the amount of ABCC2 protein and the active ezrin. Moreover, cPKC activation weakened the interaction between ABCC2 and ezrin proteins (27).

### Signaling Pathways by Which LFT Maintains the Intestinal Morphology and Health in Broilers

Our study found that LFT regulated the intestinal morphology and health in broilers *via* multifarious signaling pathways, including AGE-RAGE, ECMRI, focal adhesion, and ABC transporters. For example, *AGE* expression in the jejunal villi crypt as well as *RAGE* expression in the villi significantly enhanced the jejunal layer thickness, weight per length, and wall area (28). RAGE signaling was closely linked to intestinal permeability and inflammation. AGE-RAGE signaling and RAGE activation in the intestinal epithelium contributed to intestinal permeability and pathogenesis (29).

ECM, a vital component of the intestine, provided the structural framework and conveyed tissue-specific signals to the adjacent enterocytes. Porcine epidemic diarrhea virus (PEDV) infection resulted in extensive ECM remodeling in IECs. *SERPINE1* and *CD44*, two ECM-regulated genes, could enhance or inhibit the PEDV infection (30).

A previous research found that various signaling pathways were involved in intestinal schistosomiasis and trinitro-benzene-sulfonic acid-induced ileitis, such as ABC transporters, cell adhesion, ECMRI, antigen processing and presentation, TLR, and the response to chemical stimulus categories (31). The ABC-transporter mediated the cellular uptake, absorptive permeability, and intestinal absorption. For instance, the ABC-transporter-mediated efflux and the poor permeability were the major reasons for Rh2 poor absorption (32).

Fatty acid metabolism, such as fatty acid biosynthesis and elongation, might play a vital role in intestinal absorption and health. In our study, DEGs in chicken jejuna between LFT-treated groups and the control also enriched in fatty acid elongation and unsaturated fatty acids biosynthesis signaling pathways that were consistent with the report that polyunsaturated fatty acids, including oleic acid, linolenic acid, and conjugated linoleic acid (CLA), had a protective effect in the intestine morphology and health (33). In IECs, long-chain saturated fatty acids stimulated TG synthesis, and stearic acids and palmitic also stimulated phospholipid synthesis (34).

CLA potentially modulates gut microbiota and intestinal permeability. CLA increased intestinal permeability in the normal mice and obviously improved the tight junction proteins in the intestine of leptin-deficient mice (35). CLA increased the abundance of beneficial bacteria (such as *Roseburia, Dubosiella*, and *Anaerostipes*) and increased the abundance of pro-inflammatory bacteria (such as *Alistipes* and *Tyzzerella*) in eptin-deficient mice. In addition, gut microbiota was associated with intestinal permeability [39]. CLA increased the *SIgA* mRNA and SIgA protein content in the jejunal mucosa. CLA treatment significantly increased *PPAR*γ expression in jejunum as well as lymphocyte proliferation, and the percent of T lymphocytes (CD8^+^) in Peyer's node of broilers (36).

CLA addition could obviously enhance the immunity and antioxidant capacity of the intestinal mucosa in broilers. CLA supplementation at the level of 1.50% notably improved the CD8^+^ T lymphocytes percentage in the duodenal epithelium, reducing the concentration of malondialdehyde and glutathione in the duodenal mucosa of the birds infected by *Eimeria acervuline* but had no effects on the activities of catalase and superoxide dismutase (37).

## Conclusion

Taken together, signaling pathways (such as AGE-RAGE, fatty acid elongation, ECMRI, glycerophospholipid metabolism, focal adhesion, unsaturated fatty acids biosynthesis, and ABC transporters) and 29 genes (including *REG4, GJB1, KAT2A, APOA5, SERPINE2, ELOVL1, ABCC2, ANKRD9, CYP4V2*, and *PISD*) might be closely related to promoting jejuna morphology in broilers. Our observation enhances the understanding of LFT in maintaining intestinal architecture and the general health of broiler chickens.

## Data Availability Statement

The datasets presented in this study can be found in online repositories. The names of the repository/repositories and accession number(s) can be found in the article/Supplementary Material.

## Ethics Statement

The animal study was reviewed and approved by the Protocol from Anhui Science and Technology University (Bengbu, China) Institutional Animal Care and Use Committee (ECASTU-2015-P08).

## Author Contributions

BY conceived the study. XL wrote the manuscript and prepared the figures. XS, NM, LL, and ZC prepared the tables and analyzed the results. All authors contributed to the article and approved the submitted version.

## Funding

This study was funded by the Talent Introduction Program of Anhui Science and Technology University (No. DKYJ202003) and the National Natural Science Foundation of China (No. 31560642).

## Conflict of Interest

The authors declare that the research was conducted in the absence of any commercial or financial relationships that could be construed as a potential conflict of interest.

## Publisher's Note

All claims expressed in this article are solely those of the authors and do not necessarily represent those of their affiliated organizations, or those of the publisher, the editors and the reviewers. Any product that may be evaluated in this article, or claim that may be made by its manufacturer, is not guaranteed or endorsed by the publisher.
