# Supplementary material for: Mechanism of Lysoforte in Improving Jejuna Morphology and Health in Broiler Chickens
Source: Front Vet Sci. 2022 Jul 19;9:946148. doi: 10.3389/fvets.2022.946148 (PMC9343761; doi:10.3389/fvets.2022.946148)
Supplement: Supplementary file 1 [file Data_Sheet_1.zip › Supplementary Material/Supplementary Tables 1 - 10.docx]

Supplemental Table 1. Ingredients and nutrient content of basal diet (Brautigan et al, 2017)

| Items | Diets | |
| --- | --- | --- |
|  | Starter/% | Grower/% |
| Ingredients |  |  |
| Corn | 55.07 | 59.26 |
| Soybean meal 48 | 30.10 | 22.87 |
| DDGS | 3.18 | 6.00 |
| Stabilized fat | 2.50 | 4.80 |
| Poultry byproduct meal | 3.00 | 4.00 |
| Sodium chloride | 0.37 | 0.32 |
| DL Methionine | 0.28 | 0.23 |
| Soy oil | 1.50 | - |
| Limestone | 1.00 | 0.84 |
| Dicalcium Phos | 1.04 | 0.70 |
| Lysine-HCl | 0.20 | 0.21 |
| Threonine | 0.07 | 0.04 |
| Phytase | 500 ftu | 500 ftu |
| Choline chloride 60 | 0.10 | 0.10 |
| Mineral premix | 0.31 | 0.31 |
| Vitamin premix | 0.31 | 0.31 |
| Nutrient content |  | |
| Crude protein | 21.84 | 20.06 |
| Metabolic energy/Mcal/kg | 3.10 | 3.18 |
| Calcium | 0.80 | 0.74 |
| Available phosphorus | 0.35 | 0.32 |
| Threonine | 0.77 | 0.69 |
| Methionine + cysteine | 0.88 | 0.80 |
| Lysine | 1.18 | 1.05 |

Supplemental Table 2. Effects of LPC on chicken jejuna morphology

| Items | Groups | | | SEM | *P* Value |
| --- | --- | --- | --- | --- | --- |
|  | Control | LFT500 | LFT1000 |  |  |
| Villus length/μm | 661.01^b^ | 698.62^ab^ | 778.33^a^ | 28.00 | <0.001 |
| Villus width/μm | 140.10 | 157.48 | 152.80 | 6.24 | <0.13 |

Supplemental Table 3. The transcripts and genes significantly up- and down-regulated in LFT-treated jejunum

| Items | Comparisons | Total | Upregulated | Downregulated |
| --- | --- | --- | --- | --- |
| Transcripts | LFT1000 vs. control | 547 | 311 | 236 |
|  | LFT500 vs. control | 174 | 98 | 76 |
|  | LFT1000 vs. LFT500 | 454 | 388 | 66 |
| Genes | LFT1000 vs. control | 344 | 224 | 120 |
|  | LFT500 vs. control | 106 | 68 | 38 |
|  | LFT1000 vs. LFT500 | 300 | 257 | 43 |

Supplemental Table 4. The top 20 upregulated genes in the jejunum of LFT500 group compared to the control

| Gene symbol | Log_2_FC | *P* Value | Description |
| --- | --- | --- | --- |
| *RSAD2* | 3.24 | 1.03E-04 | Radical S-adenosyl methionine domain containing 2 |
| *SLBP* | 2.69 | 4.30E-06 | Stem-loop binding protein |
| *MMP1* | 2.69 | 2.06E-02 | Matrix metallopeptidase 1 (interstitial collagenase) |
| *FAM188A* | 2.10 | 4.99E-05 | Family with sequence similarity 188 member A |
| *SAMD9L* | 1.99 | 4.45E-04 | Sterile alpha motif domain containing 9-like |
| *SERPINB2* | 1.86 | 2.48E-02 | Serpin peptidase inhibitor, clade B (ovalbumin), member 2 |
| *REG4* | 1.75 | 4.21E-02 | Regenerating islet-derived family, member 4 |
| *OASL* | 1.68 | 1.03E-05 | 2'-5'-oligoadenylate synthetase-like |
| *CMPK2* | 1.68 | 1.07E-05 | Cytidine/uridine monophosphate kinase 2 |
| *LOC415756* | 1.62 | 2.84E-04 | Uncharacterized LOC415756 |
| *LOC420108* | 1.62 | 5.19E-04 | Uncharacterized LOC420108 |
| *ABHD3* | 1.62 | 4.32E-02 | Abhydrolase domain containing 3 |
| *GJB1* | 1.60 | 3.99E-02 | Gap junction protein, beta 1, 32kda |
| *USP18* | 1.59 | 3.89E-04 | Ubiquitin specific peptidase 18 |
| *IFIH1* | 1.58 | 2.77E-04 | Interferon induced, with helicase C domain 1 |
| *KAT2A* | 1.55 | 9.15E-06 | K(lysine) acetyltransferase 2A |
| *TLR3* | 1.54 | 3.03E-06 | Toll-like receptor 3 |
| *LOC422513* | 1.54 | 1.21E-03 | Hect domain and RLD 4-like |
| *ZC3HAV1* | 1.51 | 7.15E-07 | Zinc finger CCCH-type, antiviral 1 |
| *DHX58* | 1.43 | 8.94E-06 | DEXH-box helicase 58 |

Supplemental Table 5. The top 20 genes downregulated in the jejunum of LFT500 group compared to the control

| Gene symbol | Log_2_FC | *P* Value | Description |
| --- | --- | --- | --- |
| *AVD* | -2.56 | 3.62E-02 | Avidin |
| *LOC426100* | -2.51 | 9.60E-03 | Similar to LOC402959 protein |
| *ALCAM* | -1.82 | 7.33E-04 | Activated leukocyte cell adhesion molecule |
| *IL8L2* | -1.79 | 2.04E-02 | Interleukin 8-like 2 |
| *CHKA* | -1.66 | 4.26E-02 | Choline kinase alpha |
| *MACROD2* | -1.64 | 6.94E-04 | MACRO domain containing 2 |
| *STK38L* | -1.64 | 3.07E-03 | Serine/threonine kinase 38 like |
| *CCDC88C* | -1.63 | 1.51E-02 | Coiled-coil domain containing 88C |
| *ANGPT1L* | -1.61 | 2.03E-02 | Angiopoietin-related protein 1-like |
| *FLRT3* | -1.60 | 2.78E-02 | Fibronectin leucine rich transmembrane protein 3 |
| *JCHAIN* | -1.56 | 3.65E-02 | Joining chain of multimeric iga and igm |
| *SIPA1L2* | -1.46 | 1.32E-02 | Signal-induced proliferation-associated 1 like 2 |
| *KIAA1211* | -1.45 | 3.10E-05 | Kiaa1211 |
| *EHBP1* | -1.39 | 2.79E-03 | EH domain binding protein 1 |
| *SPTBN5* | -1.33 | 7.35E-03 | Spectrin, beta, non-erythrocytic 5 |
| *ANGPTL6* | -1.33 | 3.53E-02 | Angiopoietin-like 6 |
| *LOC420486* | -1.31 | 1.82E-02 | Mycocerosic acid synthase-like |
| *MAP7D3* | -1.27 | 3.97E-03 | MAP7 domain containing 3 |
| *SOCS3* | -1.25 | 4.47E-02 | Suppressor of cytokine signaling 3 |
| *PIK3CB* | -1.22 | 5.41E-03 | Phosphatidylinositol-4,5-bisphosphate 3-kinase, catalytic subunit beta |

Supplemental Table 6. The top 20 genes upregulated in the jejunum of LFT1000 group compared to the control

| Gene symbol | Log_2_FC | *P* Value | Description |
| --- | --- | --- | --- |
| TMPRSS15 | 3.49 | 5.93E-03 | Transmembrane protease, serine 15 |
| MT3 | 2.84 | 1.84E-02 | Metallothionein 3 |
| LOC427933 | 2.54 | 3.85E-02 | Sulfotransferase 6B1-like |
| CYP1A1 | 2.53 | 1.08E-02 | Cytochrome P450, family 1, subfamily A, polypeptide 1 |
| CYP1A4 | 2.52 | 2.71E-02 | Cytochrome P450 1A4 |
| SPARC | 2.47 | 1.26E-03 | Secreted protein, acidic, cysteine-rich (osteonectin) |
| MGP | 2.41 | 3.02E-03 | Matrix Gla protein |
| SLC22A13 | 2.38 | 4.99E-03 | Solute carrier family 22 (organic anion transporter), member 13 |
| ADH1C | 2.32 | 2.12E-02 | Alcohol dehydrogenase 1C (class I), gamma polypeptide |
| EDNRB | 2.28 | 3.16E-03 | Endothelin receptor type B |
| TFPI2 | 2.20 | 3.71E-03 | Tissue factor pathway inhibitor 2 |
| TGFBI | 2.14 | 4.12E-03 | Transforming growth factor, beta-induced, 68kda |
| CDH11 | 2.13 | 1.11E-02 | Cadherin 11, type 2, OB-cadherin (osteoblast) |
| PLEKHC1 | 2.06 | 4.09E-03 | Pleckstrin homology domain containing, family C member 1 |
| ALDH1A3 | 2.04 | 1.90E-02 | Aldehyde dehydrogenase 1 family, member A3 |
| COL1A2 | 2.03 | 1.10E-02 | Collagen, type I, alpha 2 |
| PTER | 1.99 | 1.15E-02 | Phosphotriesterase related |
| NID1 | 1.98 | 1.57E-02 | Nidogen 1 |
| EFEMP1 | 1.98 | 1.59E-02 | EGF containing fibulin-like extracellular matrix protein 1 |
| IGFBP7 | 1.95 | 4.80E-03 | Insulin like growth factor binding protein 7 |

Supplemental Table 7. The top 20 genes down-regulated in the jejunum of LFT1000 group compared to the control

| Gene symbol | Log_2_FC | *P* Value | Description |
| --- | --- | --- | --- |
| *IL8L2* | -2.83 | 6.71E-04 | Interleukin 8-like 2 |
| *LOC428714* | -2.71 | 3.47E-03 | YrdC-like domain-containing protein |
| *AVD* | -2.68 | 2.61E-02 | Avidin |
| *B3GALT5* | -2.56 | 1.62E-03 | UDP-Gal:betaglcnac beta 1,3-galactosyltransferase, polypeptide 5 |
| *LOC422306* | -2.40 | 7.67E-03 | Uncharacterized LOC422306 |
| *IRG1L* | -2.34 | 9.46E-03 | Immunoresponsive 1 homolog (mouse)-like |
| *NOXO1* | -2.24 | 3.73E-03 | NADPH oxidase organizer 1 |
| *STK38L* | -1.88 | 7.38E-03 | Serine/threonine kinase 38 like |
| *LOC420486* | -1.83 | 3.09E-03 | Mycocerosic acid synthase-like |
| *CHKA* | -1.80 | 3.11E-03 | Choline kinase alpha |
| *FCGBP* | -1.79 | 1.03E-03 | Fc fragment of igg binding protein |
| *PDCD1LG2* | -1.77 | 2.60E-02 | Programmed cell death 1 ligand 2 |
| *SLC25A48* | -1.76 | 4.06E-02 | Solute carrier family 25 member 48 |
| *MUC2* | -1.72 | 7.40E-04 | Mucin 2, oligomeric mucus/gel-forming |
| *KCNJ15* | -1.71 | 3.54E-02 | Potassium inwardly-rectifying channel, subfamily J, member 15 |
| *KIAA1211* | -1.67 | 3.20E-06 | Kiaa1211 |
| *CPT1A* | -1.67 | 7.23E-04 | Carnitine palmitoyltransferase 1A (liver) |
| *FLRT3* | -1.67 | 2.20E-02 | Fibronectin leucine rich transmembrane protein 3 |
| *SLC7A9* | -1.62 | 1.34E-03 | Solute carrier family 7, member 9 |
| *PNISR* | -1.61 | 2.83E-03 | PNN-interacting serine/arginine-rich protein |

Supplemental Table 8. The top 20 genes upregulated in the jejunum of LFT1000 group compared to LFT500 group

| Gene symbol | Log_2_FC | *P* Value | Description |
| --- | --- | --- | --- |
| *OLFM4* | 3.71 | 6.93E-03 | Olfactomedin 4 |
| *MGP* | 3.21 | 5.65E-04 | Matrix Gla protein |
| *MT3* | 3.08 | 5.08E-04 | Metallothionein 3 |
| *JCHAIN* | 3.02 | 1.26E-03 | Joining chain of multimeric IgA and IgM |
| *COL1A2* | 2.88 | 2.23E-03 | Collagen, type I, alpha 2 |
| *ALDH1A3* | 2.86 | 3.40E-03 | Aldehyde dehydrogenase 1 family, member A3 |
| *NID1* | 2.78 | 2.55E-03 | Nidogen 1 |
| *MYH11* | 2.75 | 1.08E-02 | Myosin, heavy chain 11, smooth muscle |
| *FN1* | 2.73 | 4.09E-03 | Fibronectin 1 |
| *IGFBP7* | 2.70 | 1.17E-03 | Insulin like growth factor binding protein 7 |
| *MMP2* | 2.67 | 3.17E-03 | Matrix metallopeptidase 2 |
| *SPARC* | 2.66 | 1.40E-03 | Secreted protein, acidic, cysteine-rich |
| *CFD* | 2.63 | 4.57E-03 | Complement factor D |
| *MYLK* | 2.60 | 4.10E-03 | Myosin light chain kinase |
| *TMPRSS15* | 2.58 | 1.13E-02 | Transmembrane protease, serine 15 |
| *EDNRB* | 2.57 | 1.71E-03 | Endothelin receptor type B |
| *DCN* | 2.44 | 1.68E-03 | Decorin |
| *COL1A1* | 2.43 | 2.70E-03 | Collagen, type I, alpha 1 |
| *C5H11ORF96* | 2.41 | 3.33E-03 | Chromosome 5 open reading frame, human C11orf96 |
| *CDH11* | 2.41 | 4.77E-03 | Cadherin 11, type 2, OB-cadherin |

Supplemental Table 9. The top 20 genes downregulated in the jejunum of LFT1000 group compared to LFT500 group

| Gene symbol | Log_2_FC | *P* Value | Description |
| --- | --- | --- | --- |
| *LOC418700* | -2.94 | 1.99E-05 | Lysozyme g-like |
| *RSAD2* | -2.47 | 8.03E-04 | Radical S-adenosyl methionine domain containing 2 |
| *LOC428714* | -1.88 | 2.53E-02 | YrdC-like domain-containing protein |
| *SLBP* | -1.81 | 2.96E-05 | Stem-loop binding protein |
| *SUSD2* | -1.81 | 3.34E-02 | Sushi domain containing 2 |
| *LOC415756* | -1.77 | 2.23E-04 | Uncharacterized LOC415756 |
| *FAM188A* | -1.65 | 5.27E-04 | Family with sequence similarity 188 member A |
| *SAMD9L* | -1.60 | 1.77E-03 | Sterile alpha motif domain containing 9-like |
| *LOC422306* | -1.60 | 3.20E-02 | Uncharacterized LOC422306 |
| *SCN3B* | -1.56 | 3.97E-03 | Sodium channel, voltage-gated, type III, beta subunit |
| *LY6E* | -1.51 | 1.54E-03 | Lymphocyte antigen 6 complex, locus E |
| *FGF19* | -1.50 | 2.56E-03 | Fibroblast growth factor 19 |
| *MATN2* | -1.36 | 1.34E-02 | Matrilin 2 |
| *COL9A3* | -1.36 | 1.59E-02 | Collagen, type IX, alpha 3 |
| *B4GALT6* | -1.35 | 2.65E-02 | Beta-1,4-galactosyltransferase 6 |
| *PDE9A* | -1.33 | 1.11E-02 | Phosphodiesterase 9A |
| *TRANK1* | -1.30 | 9.84E-03 | Tetratricopeptide repeat and ankyrin repeat containing 1 |
| *IFIT5* | -1.26 | 8.47E-04 | Interferon-induced protein with tetratricopeptide repeats 5 |
| *IFIH1* | -1.23 | 2.45E-03 | Interferon induced, with helicase C domain 1 |
| *ENDOV* | -1.22 | 2.12E-02 | Endonuclease V |

Supplemental Table 10. The common DEGs in the two comparisons (LFT500 and LFT1000 vs. control)

| Gene symbol | Full name | Functions |
| --- | --- | --- |
| *REG4* | Regenerating islet-derived family, member 4 | *REG4* is related to adhesion, calcium ion binding and heparin binding. |
| *GJB1* | Gap junction beta-1 protein | *GJB1* protein is a member of the gap junction protein family which facilitate the transfer of ions and small molecules between cells. |
| *KAT2A* | Histone acetyltransferase kat2a | KAT2A is a histone acetyltransferase (HAT) that functions primarily as a transcriptional activator. It also functions as a repressor of NF-kappa-B. |
| *APOA5* | Apolipoprotein A5 | *APOA5* plays an important role in regulating the plasma triglyceride levels, a major risk factor for coronary artery disease. |
| *SERPINE2* | Serpin family E member 2 | *SERPINE2* encodes a member of the serpin family of proteins, a group of proteins that inhibit serine proteases. Thrombin, urokinase, plasmin and trypsin are among the proteases that this family member can inhibit. |
| *ELOVL1* | Elongation of very long chain fatty acids protein 1 | *ELOVL1* enables fatty acid elongase activity. *ELOVL1* is involved in fatty acid biosynthetic process and sphingolipid biosynthetic process. |
| *ABCC2* | ATP-binding cassette, sub-family C | ABCC2 protein is a member of the superfamily of ATP-binding cassette (ABC) transporters. ABCC2 protein is a member of the MRP subfamily which is involved in multi-drug resistance. |
| *ANKRD9* | Ankyrin repeat domain-containing protein 9 | *ANKRD9* enables ubiquitin ligase-substrate adaptor activity and is involved in cellular copper ion homeostasis; proteasome-mediated ubiquitin-dependent protein catabolic process; and protein ubiquitination. |
| *CYP4V2* | Cytochrome P450 family 4 subfamily V member 2 | CYP4V2 is a member of the cytochrome P450 hemethiolate protein superfamily which are involved in oxidizing various substrates in the metabolic pathway, such as the metabolism of fatty acid precursors into n-3 polyunsaturated fatty acids. |
| *PISD* | Phosphatidylserine decarboxylase | PISD protein catalyzes the conversion of phosphatidylserine to phosphatidylethanolamine in the inner mitochondrial membrane. PISD protein is active in phospholipid metabolism and interorganelle trafficking of phosphatidylserine. |
| *PTGR1* | Prostaglandin reductase 1 | PTGR1 is involved in the inactivation of the chemotactic factor, leukotriene B4. PTGR1 protein specifically catalyzes the NADP+ dependent conversion of leukotriene B4 to 12-oxo-leukotriene B4. |
| *LOC431499* | Coiled-coil domain-containing protein 81 | - |
| *AKAP9* | A kinase anchor protein 9 | AKAP9 is related to cytokine signaling in immune system, RET signaling, and the binding of signaling receptor and transmembrane transporter. |
| *TET2* | Tet methylcytosine dioxygenase 2 | TET2 protein is a methylcytosine dioxygenase that catalyzes the conversion of methylcytosine to 5-hydroxymethylcytosine. *TET2* protein is involved in myelopoiesis and several myeloproliferative disorders. |
| *RALGAPA2* | Ral GTPase-activating protein subunit alpha-2 | *RALGAPA2* is involved in GTPase activity activation and the regulation of exocyst localization and protein localization. |
| *MYO1D* | myosin ID | *MYO1D* is involved in actin filament organization; early endosome to recycling endosome transport; and vesicle transport along actin filament. Located in extracellular exosome. |
| *RSRC2* | Arginine and serine rich coiled-coil 2 | *RSRC2* is related to RNA binding. An important *RSRC2* paralog is RSRP1. |
| *SOCS3* | SOCS family proteins | SOCS3 is involved in negative regulation of cytokines that signal through the JAK/STAT pathway. SOCS3 inhibits cytokine signal transduction by binding to tyrosine kinase receptors. |
| *MAP7D3* | MAP7 domain containing 3 | *MAP7D3* enables protein domain specific binding activity and is involved in actin filament organization; early endosome to recycling endosome transport; and vesicle transport along actin filament. |
| *LOC420486* | Mycocerosic acid synthase-like | - |
| *SPTBN5* | Spectrin, beta, non-erythrocytic 5 | *SPTBN5* enables several functions, including cytoskeletal protein binding activity; dynein intermediate chain binding activity; and identical protein binding activity. |
| *KIAA1211* | KIAA1211 ortholog | - |
| *SIPA1L2* | Signal-induced proliferation-associated 1-like protein 2 | *SIPA1L2* encodes a member of the signal-induced proliferation-associated 1 like family. |
| *FLRT3* | Leucine-rich repeat transmembrane protein | *FLRT3* plays a role in vascular development and cell-cell adhesion via its interaction with latrophilins expressed at the surface of adjacent cells. |
| *MACROD2* | O-acetyl-adp-ribose deacetylase macrod2 | MACROD2 protein is a deacetylase involved in removing ADP-ribose from mono-ADP-ribosylated proteins. It translocate from the nucleus to the cytoplasm upon DNA damage. |
| *STK38L* | Serine/threonine kinase 38 like | *STK38L* enables ATP binding activity; magnesium ion binding activity; and protein serine/threonine kinase activity. *STK38L* involved in intracellular signal transduction. |
| *CHKA* | Choline kinase alpha | CHKA protein is the initial enzyme in the sequence and may play a regulatory role. CHKA encoded protein also catalyzes the phosphorylation of ethanolamine. |
| *ALCAM* | Activated leukocyte cell adhesion molecule | *ALCAM* mediates both heterotypic cell-cell contacts and promotes T-cell activation and proliferation. |
| *AVD* | Avidin precursor | *AVD* enables ATP binding activity; magnesium ion binding activity; and protein serine/threonine kinase activity. *AVD* is involved in intracellular signal transduction. |
| *COL1A1* | Collagen type I alpha 1 chain | *COL1A1* is a fibril-forming collagen found in most connective tissues and is abundant in bone, cornea, dermis and tendon. |
| *COL4A2* | Collagen type I alpha 2 chain | *COL1A2* encodes the pro-alpha2 chain of type I collagen whose triple helix comprises two alpha1 chains and one alpha2 chain. |
| *IL8L2* | Interleukin 8-like 2 | *IL8L2* may be an autocrine factor that promotes the growth of fibroblasts and is involved in the neoplastic transformation of fibroblasts by v-Src. |
